# Activation of C-reactive protein proinflammatory phenotype in the blood retinal barrier *in vitro*: implications for age-related macular degeneration

**DOI:** 10.18632/aging.103655

**Published:** 2020-07-16

**Authors:** Sara Romero-Vázquez, Alfredo Adán, Marc Figueras-Roca, Victor Llorenç, Mark Slevin, Gemma Vilahur, Lina Badimon, Andrew D Dick, Blanca Molins

**Affiliations:** 1 Group of Ocular Inflammation, Clinical and Experimental Studies, Institut d’Investigacions Biomèdiques Agustí Pi i Sunyer (IDIBAPS), Hospital Clínic de Barcelona, Barcelona, Spain; 2 Department of Life Sciences, Manchester Metropolitan University, Manchester, UK; 3 Cardiovascular Research Center-ICCC, Hospital de la Santa Creu i Sant Pau, IIB-Sant Pau, CiberCV, Institute Carlos III, Barcelona, Spain; 4 Academic Unit of Ophthalmology, School of Clinical Sciences and School of Cellular and Molecular Medicine, University of Bristol, Bristol, UK; 5 National Institute for Health Research (NIHR) Biomedical Research Centre at Moorfields Eye Hospital and University College London Institute of Ophthalmology, London, UK

**Keywords:** age-related macular degeneration, retinal pigment epithelium, inflammation, C-reactive protein

## Abstract

The retinal pigment epithelium (RPE) is considered one of the main targets of age-related macular degeneration (AMD), the leading cause of irreversible vision loss among the ageing population worldwide. Persistent low grade inflammation and oxidative stress eventually lead to RPE dysfunction and disruption of the outer blood-retinal barrier (oBRB). Increased levels of circulating pentameric C-reactive protein (pCRP) are associated with higher risk of AMD. The monomeric form (mCRP) has been detected in drusen, the hallmark deposits associated with AMD, and we have found that mCRP induces oBRB disruption. However, it is unknown how mCRP is generated in the subretinal space. Using a Transwell model we found that both pCRP and mCRP can cross choroidal endothelial cells and reach the RPE *in vitro* and that mCRP, but not pCRP, is able to cross the RPE monolayer in ARPE-19 cells. Alternatively, mCRP can originate from the dissociation of pCRP in the surface of lipopolysaccharide-damaged RPE in both ARPE-19 and primary porcine RPE lines. In addition, we found that the proinflammatory phenotype of mCRP in the RPE depends on its topological localization. Together, our findings further support mCRP contribution to AMD progression enhancing oBRB disruption.

## INTRODUCTION

Age-related macular degeneration (AMD) is the primary cause of irreversible vision loss among the ageing population worldwide. The number of people with AMD worldwide is expected to reach 196 million in 2020, increasing to 288 million in 2040 [1]. AMD is a degenerative and progressive disease involving multiple genetic and environmental factors, age being the primary risk factor.

The retinal pigment epithelium (RPE) monolayer is believed to be among the initial targets of early disease. AMD presents RPE cell abnormalities, disruption of the outer blood-retinal-barrier (oBRB), and degeneration of photoreceptors, which require a normally functioning RPE to survive [2, 3]. Prior research has implicated strong roles for inflammation, oxidative stress, lipid abnormalities, and RPE dysfunction in AMD pathobiology, but their precise mechanisms and their relative contribution are unclear [4]. A multitude of systemic changes occur with ageing that contribute to the initiation and development of inflammation. Indeed, the immune system of elderly individuals is characterized by a basal systemic inflammatory state [5].

Altered immune responses are thought to contribute to the dry AMD phenotype. Parainflammation is a low-grade cytoprotective adaptation to local stress that is intermediate between immune-mediated homeostasis and chronic inflammation that maintains cellular and tissue function. Loss of parainflammation control contributes to AMD by invoking a chronic, heightened immune response that causes tissue destruction [6–8]. Histochemical and proteomic analysis of ocular drusen, the hallmark deposits of AMD, have shown that these deposits contain inflammatory proteins and complement components that mediate local inflammation [9, 10]. Furthermore, the strongest genetic risk factor for AMD known to date is a common polymorphism in the *complement factor H* (*CFH*) gene (c.1277T > C, p.Tyr402His), a gene essential for the regulation of complement activation [11, 12].

C-reactive protein (CRP), a prototypical acute-phase reactant, is an active regulator of the innate immune system. Among the multiple functions ascribed to CRP are activation of the classical complement pathway and inactivation of the alternative pathway [13]. CRP is considered to be a serum biomarker for chronic inflammation, heart disease and, more recently, also AMD [14, 15]. In plasma, CRP typically exists as a cyclic, disk-shaped pentamer (pentameric CRP, pCRP) composed of five noncovalently linked subunits of 23 kDa [16]. However, pCRP can undergo dissociation into its subunits, acquiring distinct biological functions. Oxidative stress and bioactive lipids from activated or damaged cells can dissociate pCRP into its 23-kDa subunits [17–19] through a mechanism that is dependent on lysophosphatidylcholine (LPC) exposure after phospholipase A_2_ activation [20]. This alternative conformation of CRP, termed monomeric CRP (mCRP), has different antigenicity-expressing neoepitopes than pCRP and represents the tissue-based insoluble form of CRP. Unlike pCRP, mCRP displays a proinflammatory phenotype in several cell types [21–23].

mCRP has been identified in ocular drusen and other subepithelial deposits [24, 25], as well as in the choroid, and we have shown that mCRP, but not pCRP, contributes to oBRB disruption *in vitro* [26]. Moreover, we also showed that the “non-risk” Factor H (FH) variant can effectively bind to mCRP to dampen mCRP pro-inflammatory activity [27]. Notably, FH from AMD patients carrying the risk polymorphism for AMD shows an impaired binding to mCRP and, therefore, its proinflammatory effects remain unrestrained [28]. In line with these findings, data demonstrates that mCRP is the more abundant form of CRP in human RPE-choroid [29], and that mCRP levels are elevated in individuals with the high-risk *CFH* genotype [29, 30].

If mCRP pro-inflammatory capacity is unrestrained in AMD and particularly in high risk patients, then we need to determine how mCRP is generated or accumulates in the subretinal space as there is no CRP transcription in the retinal tissue [30, 31]. In addition, it is also unclear whether mCRP-induced barrier disruption depends on its topological localization.

## RESULTS

### Choroidal endothelial cells allow diffusion of CRP isoforms

**Figure 1:**
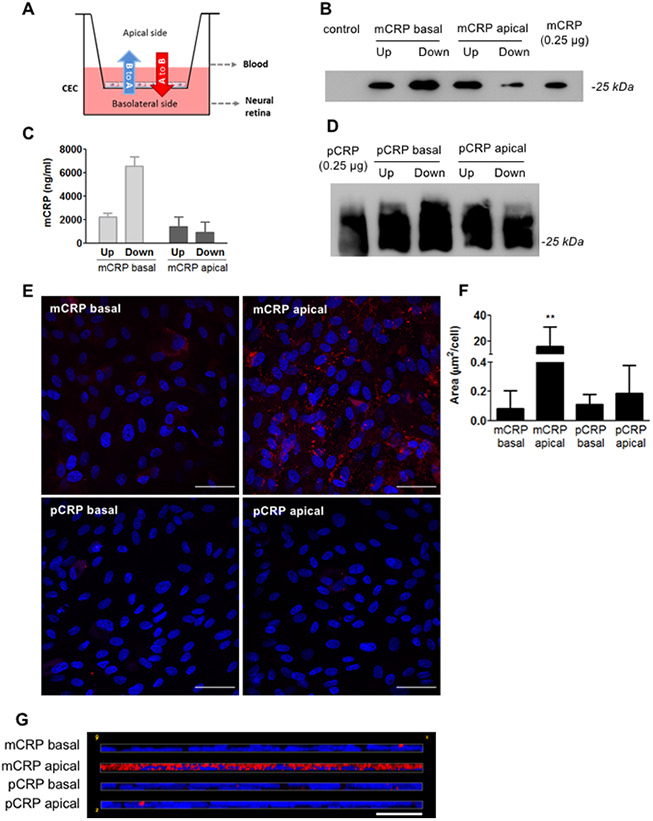
**CRP isoforms are able to cross CECs.** (**A**) Experimental setup. CRP (10 μg/ml) was added to either the apical or basolateral chamber of the Transwell for 48h, mimicking blood vessel lumen and RPE, respectively. The presence of CRP in the opposite chamber where it was added was determined by Western blot and ELISA, and CRP bound to the cell surface was determined by immunofluorescence. (**B**) Western blot of mCRP present in apical (Up) and basolateral (Down) chamber (N=4). (**C**) ELISA of mCRP (ng/ml) from apical (Up) and basolateral (Down) supernatants. Values are expressed as mean ± SD (N=3). (**D**) Western-blot of pCRP present in apical (Up) and basolateral (Down) supernatants (N=5). (**E**) Immunofluorescence of CRP (red) stained with monoclonal antibodies against mCRP (3H12) or pCRP (1C6). Nuclei stained with DAPI. Scale bar = 50 μm (N=6). (**F**) Quantification of CRP binding measured as stained area divided by the number of cells per image (μm^2^/cell). Results are expressed as mean area (μm^2^/cell) ± SD. Statistical analysis was performed by One-Way ANOVA and Tukey’s posthoc. **P<0.01 vs. all conditions. (**G**) Reconstruction of x-z sections with a 0.3 μm z axis step of immunofluorescence images. Images shown are representative of six independent experiments.

We first interrogated whether circulating CRP could reach the subretinal space using a Transwell model, in which confluent monolayers of primary porcine choroidal endothelial cells (CECs) were grown on porous filters with their apical and basolateral surfaces exposed to separate chambers (Figure 1A). Addition of mCRP to the apical chamber that mimics blood vessel lumen (A to B red arrow in Figure 1A) resulted in CRP diffusion into the basolateral chamber (tissue side) as Western blot (Figure 1B) and ELISA (Figure 1C) of the culture media of the different compartments revealed the presence of mCRP in both chambers. Similarly, pCRP was able to reach the abluminal side of the CEC monolayer, as seen by Western blot (Figure 1D). CRP isoforms were also able to reach the apical chamber when added in the abluminal compartment (B to A blue arrow in Figure 1A), suggesting bidirectional diffusion of the proteins. Immunofluorescence imaging showed that mCRP delivered to the apical compartment was extensively bound to the CEC surface compared to pCRP and to CRP (either mCRP or pCRP) delivered in the basolateral chamber (Figure 1E–1G).

### Diffusion of CRP across the RPE

**Figure 2:**
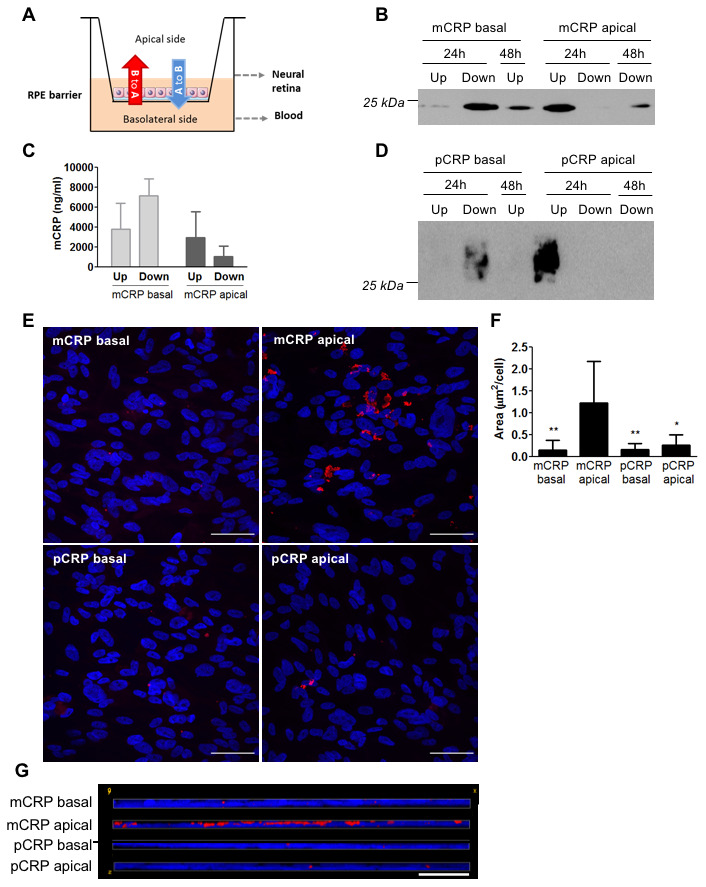
**Diffusion of CRP isoforms across ARPE-19 cells.** (**A**) Experimental setup. CRP (10 μg/ml) was added to either the apical or basolateral chamber of Transwell for 48h, mimicking neural retina and choriocapillaris, respectively. The presence of CRP in the opposite chamber where it was added was determined by Western blot and ELISA, and CRP bound to the cell surface was determined by immunofluorescence. (**B**) Western blot of mCRP present in apical (Up) and basolateral (Down) supernatants after 24 and 48 hours of treatment (N=4). (**C**) ELISA of mCRP (ng/ml) from apical (Up) and basolateral (Down) supernatants 48 hours after treatment. Values are expressed as mean ± SD (N=3). (**D**) Western blot of pCRP present in apical (Up) and basolateral (Down) supernatants after 24 and 48 hours of treatment (N=4). (**E**) Immunofluorescence of CRP (red) stained with monoclonal antibodies against mCRP (3H12) or pCRP (1C6). Nuclei stained with DAPI. Scale bar = 50 μm (N=6). (**F**) Quantification of CRP binding measured as stained area divided by the number of cells per image (μm^2^/cell). Results are expressed as mean area (μm^2^/cell) ± SD. Statistical analysis was performed by One-Way ANOVA and Tukey’s posthoc. * P<0.05, ** P<0.01 vs. mCRP apical. (**G**) Reconstruction of x-z sections with a 0.3 μm z axis step of immunofluorescence images. Images shown are representative of six independent experiments.

Given that CRP isoforms were able to cross the CEC monolayer in our *in vitro* model, we next evaluated whether CRP isoforms could also reach the subretinal space and cross the RPE, using the Transwell model. In this scenario the basolateral side of the RPE monolayer represents the Bruch’s membrane/choriocapillaris side, whereas the apical side represents the subretinal space (Figure 2A). Western blot experiments revealed that mCRP was able to diffuse across ARPE-19 cell monolayer, as it was present in the apical chamber when added in the basolateral chamber (B to A, red arrow in Figure 2A). Diffusion of mCRP was greater at 48 hours compared to 24 hours (Figure 2B). We also detected the presence of mCRP by ELISA 48 hours after treatment in the opposite chamber where it was added (Figure 2C). By contrast, pCRP did not seem to cross the ARPE-19 monolayer. Western blot experiments showed that pCRP was not present in the supernatant of the opposite chamber where it was added, neither at 24h nor at 48h after treatment (Figure 2D). Immunofluorescence imaging showed that mCRP delivered to the apical compartment was extensively bound to the ARPE-19 cell monolayer compared to pCRP and to CRP (either mCRP or pCRP) delivered in the basolateral chamber (Figure 2E–2G).

**Figure 3:**
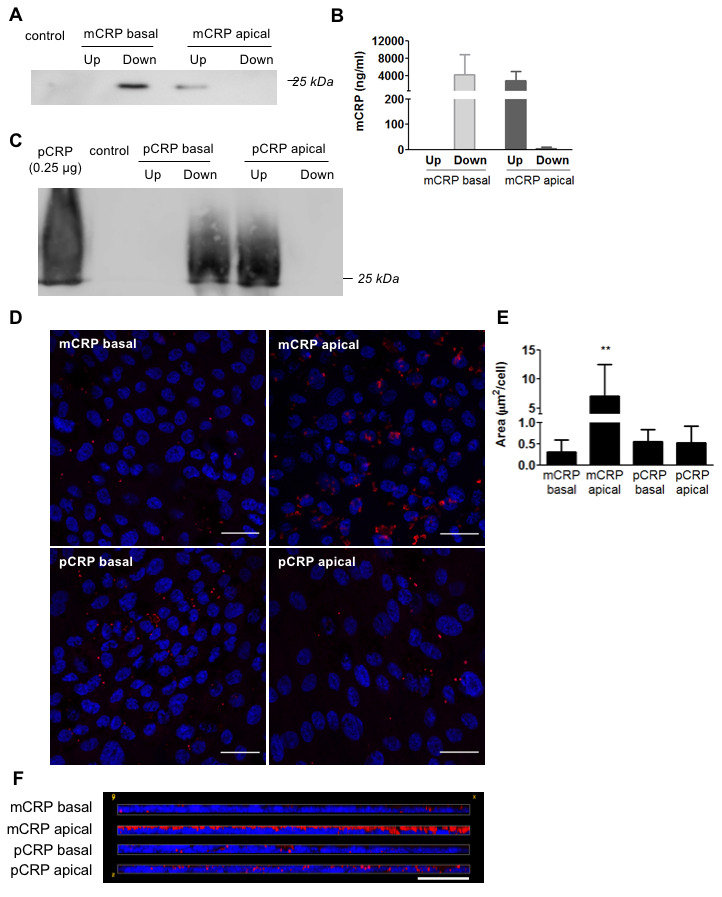
**Diffusion of CRP isoforms across primary porcine RPE cells.** (**A**) Western blot of mCRP present in apical (Up) and basolateral (Down) supernatants 48 hours after addition of mCRP (N=4). (**B**) ELISA of mCRP (ng/ml) from apical (Up) and basolateral (Down) supernatants. Values are expressed as mean ± SD (N=5). (**C**) Western blot of pCRP present in apical (Up) and basolateral (Down) supernatants 48 hours after treatment (N=3). (**D**) Immunofluorescence of CRP (red) stained with monoclonal antibodies against mCRP (3H12) or pCRP (1C6). Nuclei stained with DAPI. Scale bar = 30 μm (N=3). (**E**) Quantification of CRP binding measured as stained area divided by the number of cells per image (μm^2^/cell). Results are expressed as mean area (μm^2^/cell) ± SD. Statistical analysis was performed by One-Way ANOVA and Tukey’s posthoc. **P<0.01 vs. all conditions. (**F**) Reconstruction of x-z sections with a 0.3 μm z axis step of immunofluorescence images. Images shown are representative of three independent experiments.

We then tested whether mCRP was also able to cross porcine primary RPE cells. Primary RPE cells represent a healthier and younger RPE than ARPE-19 cells, as they show more than 5 times higher TEER values (Supplementary Figure 1D, 1E). Interestingly, unlike ARPE-19 cells, primary porcine RPE cells did not allow mCRP diffusion as mCRP was not detected in the opposite chamber where it was added, neither in Western blot (Figure 3A) nor ELISA (Figure 3B). As expected, pCRP was also unable to cross the RPE monolayer (Figure 3C). Immunofluorescence imaging showed similar results to those with ARPE-19; mCRP delivered to the apical compartment was extensively bound to the RPE cell monolayer compared to pCRP and to CRP (either mCRP or pCRP) delivered in the basolateral chamber (Figure 3D–3F).

### Damaged RPE dissociates pCRP into mCRP

**Figure 4:**
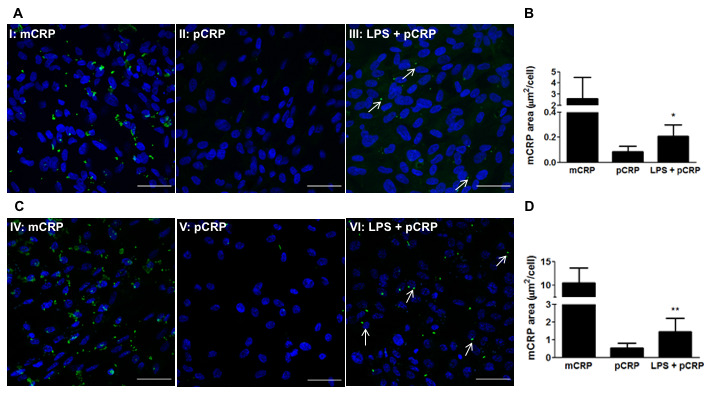
**LPS-induced inflammation promotes CRP dissociation in RPE cells.** RPE cells were treated with 100 μg/mL LPS for 24h before adding pCRP. After 24h, RPE cells were treated with pCRP for 48h and the presence of mCRP on the surface of RPE cells was measured by immunofluorescence. mCRP immunostaining of ARPE-19 (**A**) and primary porcine RPE (**B**) cells treated with 10 μg/ml mCRP for 48h (I, IV), 25 μg/ml pCRP for 48h (II, V), or 100 μg/ml LPS 24h before treatment with 25 μg/ml pCRP for 48h (III, VI). Arrows point mCRP dissociated from pCRP on RPE surface. Nuclei stained with DAPI. Scale bar = 50 μm. Images shown are representative of three independent experiments. (**C**, **D**) Quantification of CRP dissociation measured as stained area with the monoclonal antibody 3H12 against mCRP (green) divided by the number of cells per image (μm^2^/cell). Results are expressed as mean area (μm^2^/cell) ± SD (N=3). Statistical analysis was performed by student t-test. *P<0.05 vs. pCRP.

Given that pCRP is able to cross the CEC monolayer and reach the subepithelial space *in vitro*, we next studied whether pCRP could dissociate into its monomeric subunits within the RPE. It has been previously described that LPS-induced inflammation induces CRP dissociation in the cremaster muscle [20], and therefore we studied whether LPS-induced inflammation could also lead to CRP dissociation in RPE cells. RPE cells were treated with 100 μg/mL LPS for 24h before adding pCRP. After 24h, RPE cells were treated with pCRP for 48h and the presence of mCRP on the surface of RPE cells was measured by immunofluorescence. As observed in Figure 4, LPS-induced inflammation triggered pCRP dissociation into mCRP in both ARPE-19 (Figure 4A, 4B) and primary porcine RPE cells (Figure 4C, 4D). Altogether these results show that mCRP present in drusen and in the subretinal space may either arrive from the choroidal circulation or it may originate from local dissociation of pCRP in damaged RPE.

### Topological localization of mCRP determines the impact on barrier disruption in RPE cells

**Figure 5:**
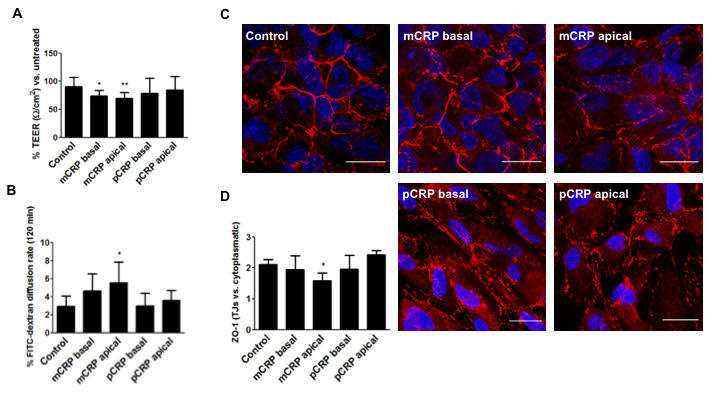
**mCRP induces barrier disruption in ARPE-19 cells in a polarized manner.** ARPE-19 cells were treated with CRP isoforms for 48h either from the apical side or the basolateral chamber and TEER (**A**) and paracellular permeability as determined by FITC-dextran diffusion rate (**B**) was determined. (**C**) Cells were then fixed and immunostained with anti ZO-1 (red) and DAPI (blue). Images shown are representative of four independent experiments. Scale bar = 20 μm. (**D**) Quantification of ZO-1 at the TJs expressed as relative (intercellular/cytoplasmic) ZO-1 distribution. Values are expressed as mean ± SD and statistical analysis was performed by one-way ANOVA and Dunnett´s posthoc analysis (N=4). * P<0.05, ** P<0.01 vs. control.

Next, we evaluated whether mCRP-induced barrier disruption depended on the topological localization of mCRP. For this purpose, ARPE-19 cells grown on inserts for at least 3 weeks were treated with CRP isoforms (10 μg/mL) either in the apical or basolateral compartment for 48h. As expected, mCRP delivered in the apical chamber, significantly decreased TEER values (Figure 5A). This observation was accompanied by an increase in paracellular permeability (Figure 5B) and an increased ZO-1 disorganization (Figure 5C, 5D, Supplementary Figure 3). Interestingly, abluminal treatment of mCRP also induced a significant decrease in TEER values.

**Figure 6:**
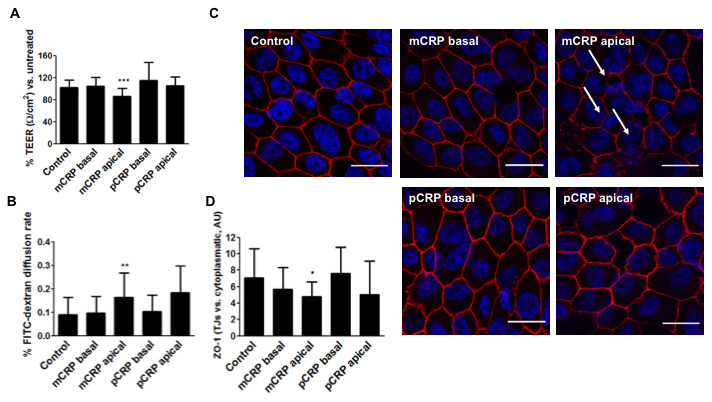
**mCRP induces barrier disruption in primary porcine RPE cells in a polarized manner.** Primary porcine RPE cells were treated with CRP isoforms for 48h either from the apical side or the basolateral chamber and TEER (**A**) and paracellular permeability as determined by FITC-dextran diffusion rate (**B**) was determined. (**C**) Cells were then fixed and immunostained with anti ZO-1 (red) and DAPI (blue). Images shown are representative of four independent experiments. Arrows show disruption of ZO-1. Scale bar = 20 μm. (**D**) Quantification of ZO-1 at the TJs expressed as relative (intercellular/cytoplasmic) ZO-1 distribution. Values are expressed as mean ± SD and statistical analysis was performed by one-way ANOVA and Dunnett´s posthoc analysis (N=6). * P<0.05, ** P<0.01, *** P<0.0001 vs. control.

We then aimed to replicate the experiments in primary porcine RPE cells, which are less permeable to mCRP diffusion. As seen in Figure 6, apical treatment of mCRP, but not pCRP, induced barrier disruption also in primary RPE cells as seen by significant decrease in TEER (Figure 6A), increase in paracellular permeability (Figure 6B) and increased ZO-1 disorganization (Figure 6C, 6D, Supplementary Figure 4). However, when mCRP was delivered into the abluminal compartment it failed to induce barrier disruption, showing that mCRP-induced barrier disruption depends on its topological localization.

## DISCUSSION

The present study aimed to understand the relative contribution of mCRP to the pathophysiology of AMD. Our *in vitro* work demonstrates that mCRP is capable of traversing through choroidal vascular endothelium and across RPE. Although no direct *in vivo* correlate, the data suggest that mCRP can reach the subretinal space. Alternatively, mCRP may derive from the dissociation of pCRP on the surface of damaged RPE. Moreover, we found that the proinflammatory phenotype of mCRP in the RPE depends on its topological localization. Together the data continues to build the evidence of mCRP accentuation of AMD pathology and detriment to RPE health.

CRP is mainly produced in the liver and, although extrahepatic synthesis has been reported in some tissues, no evidence of *CRP* gene transcription has been detected in the retinal tissue [30, 31]. This indicates that the systemic circulation is the main source of CRP in the sub-RPE deposits. Indeed, we observed that both CRP isoforms, at clinically relevant concentrations, are able to cross CECs from their apical side -simulating blood side- and reach the basolateral side of the endothelium -simulating the subepithelial side- in a Transwell model (Figure 1). The choroidal endothelium is fenestrated, which allows the movement of macromolecules and nutrients to nourish the RPE cells [32]. Although mCRP could extravasate the endothelium and reach the subepithelial space, we observed by immunofluorescence that most mCRP was retained in the apical side of the endothelium. Indeed, mCRP promiscuously interacts with a variety of immunoglobulins and other proteins [33]. Thus, we tested whether mCRP in the subepithelial space could also originate from the dissociation of pCRP in the RPE. Using the approach of Thiele et al. [20], we found that LPS-induced inflammation lead to pCRP dissociation also in RPE cells (Figure 4). Mechanistically, this process is dependent on exposure of LPC, a bioactive lipid that is generated after phospholipase A_2_ activation on activated cell membranes [20]. Chirco et al. found that mCRP is predominantly localized in the choriocapillaris and Bruch´s membrane [29] and a previous work by *Johnson and colleagues*, looking at total CRP, showed that CRP was more abundant in donor eyes with the high-risk *CFH* polymorphism compared to age-matched controls, especially in regions containing drusen-like deposits [30]. A similar study compared differences in total CRP immunoreactivity in the retina based on AMD status and found that early and wet AMD eyes had higher levels of CRP compared to controls and that CRP was primarily detected into the BM [34]. In their work, Chirco et al. also showed that mCRP exerts an inflammatory effect on CEC, as it increases CEC migration and paracellular permeability and upregulates inflammatory gene expression including *ICAM1*, suggesting a role for mCRP in promoting inflammation in the choroid.

Besides the proinflammatory effect in the choroid, we have previously demonstrated that clinically relevant concentrations of mCRP induce barrier disruption and have a proinflammatory effect in RPE *in vitro* and potential for driving angiogenesis [26, 27]. With respect to angiogenesis and neovessels, in the context of vascular disease, CRP inhibits VEGF production and angiogenesis [35, 36]. Conversely, others have shown CRP upregulates VEGF expression in adipose-derived stem cells and in monocytes [37, 38]. mCRP has been localized around newly formed microvessels in carotid artery plaques and in peri-infarct regions after an acute ischemic stroke [39, 40], promoting angiogenesis and inducing inflammation [41]. One notion is understanding of mCRP role is context dependent and in vivo studies are required to determine the contribution of mCRP to neovessel formation in the context of AMD, either via VEGF dependent or -independent mechanisms. On the other hand, Lauer et al. showed that mCRP binds necrotic RPE cells and that complement regulation at necrotic cell lesions is impaired by the FH His402 risk variant [17]. In these studies, RPE cells were stimulated with mCRP from the apical side. However, given the polarized nature of RPE cells it could be possible that the proinflammatory effect of mCRP on RPE cells depends on its topological localization. Our results showed a polarized stimulation of mCRP on barrier disruption in RPE cells, in both ARPE-19 and primary porcine RPE cells. The addition of mCRP to the apical side of RPE cells resulted in a significantly greater barrier disruption -decreased TEER, increased permeability and disrupted membrane ZO-1- than the addition of mCRP to the basolateral side. This effect was more pronounced in primary RPE cells, where mCRP had no effect on barrier disruption when added from the basolateral side, than in ARPE-19 cells. Indeed, we observed by immunofluorescence a preferential binding of mCRP to the apical side (Figures 2, 3). The polarized proinflammatory effect of mCRP has been already observed in endothelial cells using a similar approach [42]. These observations could be due to a polarized distribution of the surface sensors for mCRP in the cell surface.

The receptors that mediate mCRP activities have not been fully characterized. In human neutrophils, mCRP binds FcγRIII (CD16) [21]. However, functional blockade of CD16 showed only a slight attenuation of mCRP-induced activation in RPE and endothelial cells (ECs) [27, 43]. Instead, in ECs, lipid raft microdomains seem to be the major sensors for mCRP [44]. Therefore, it could be speculated that mCRP interacts with RPE cells through lipid raft microdomains. Nevertheless, unlike many other surface receptors in epithelial tissues, caveolae seem to have a bipolar distribution in RPE cells [45]. The increased immunoreactivity of mCRP when added to the apical side could be also attributed to the presence of the Transwell filter in the basolateral side that could hamper the binding of mCRP to the basolateral side. This could also prevent mCRP to induce barrier disruption when added from the basolateral side. However, we used filters with a pore size of 0.4 μm, big enough to allow mCRP to reach the basolateral side of the RPE cells. It could be also possible that the receptors on the apical side of the cells have a greater affinity for mCRP than those in the basolateral side. However, we observed a similar pattern with different cell types (CEC, ARPE-19, and primary RPE), and therefore it is likely that our observations are mainly the consequence of mCRP settling on the cell surface.

Given that mCRP seems to induce higher barrier disruption when present in the apical side of RPE cells, we tested whether mCRP could cross the RPE and reach the apical side when added in the basolateral side. RPE cells are critical for oBRB function, enabling selective transport of molecules in and out of the retina to preserve its immune privilege [7]. We found that pCRP was unable to reach the apical side -representing the subretinal space- of RPE cells. However, mCRP was able to reach the apical side only in ARPE-19 cells, but not in primary porcine RPE cells (Figures 2, 3). ARPE-19, is a spontaneously arising RPE cell line that behaves in many ways like primary RPE cultures as they exhibit barrier functions mediated by tight junctions and secrete cytokines. However, they exhibit reduced TEER [46]. Indeed, ARPE-19 cells are commonly used for studying oxidative stress and cell signaling in AMD because they exhibit features of aged RPE [47]. Thus, the fact that ARPE-19 cells but not primary RPE cells allow mCRP diffusion to the apical side suggest that mCRP could reach the subretinal space when the RPE is damaged. These findings may explain why mCRP had some effect on barrier disruption on ARPE-19 cells but not in primary RPE cells when added from the basolateral side. As such, some mCRP may have crossed the ARPE-19 monolayer reaching the apical side, thereby inducing barrier disruption.

Our current work suggests a plausible mechanism by which mCRP may contribute to RPE dysfunction and AMD progression: the serum-associated isoform of CRP (pCRP), would reach the oBRB by diffusion through CECs from the choroidal circulation. Once there, it would undergo dissociation into mCRP via LPC exposed in RPE surface in an inflammatory microenvironment. Alternatively, mCRP could also be generated elsewhere -although in small amounts- or in the surface of CECs before reaching the oBRB. The mCRP in the subepithelial space could reach the apical side of a damaged RPE and amplify the inflammation further disrupting the RPE barrier integrity.

The present work carries some limitations. Firstly, we used a simple model that did not incorporate a proper analogue of the Bruch’s membrane. We used fibronectin to grow CECs, laminin to grow primary porcine RPE cells, and ARPE-19 cells were grown without any protein coating in the Transwell filters. Secondly, we did not use RPE cells derived from inducible pluripotent stem cells which would have added more translatability to our work. However, we used two different models of RPE to understand how mCRP contributes to AMD progression, albeit in an *in vitro* setting.

In summary, our findings further support mCRP direct contribution to progression of AMD, at least at the RPE level. The topological experiments elicit that mCRP is proinflammatory when present on the apical side of the RPE. However, mCRP is likely to only reach the apical side of the RPE in compromised RPE health and where barrier functions are compromised. Thus, a plausible scenario would infer that, in the presence of an already aged/damaged RPE, mCRP reaches the apical side of the RPE to amplify the proinflammatory microenvironment and enhance barrier disruption. With respect to previous findings, this pathologic mechanism will be more prevalent in patients carrying the *FH* risk polymorphism for AMD, where mCRP proinflammatory effects remain unrestrained [28].

## MATERIALS AND METHODS

### CRP isoforms

High purity human pCRP (Calbiochem) was stored in 10 mM Tris, 140 mM NaCl buffer (pH 8.0) containing 2 mM CaCl_2_ to prevent spontaneous formation of mCRP from the native pentamer. mCRP was obtained by urea chelation from purified human CRP as previously described [23]. Briefly, pCRP at 1 mg/mL was chelated with 10 mM ethylene diaminetetraacetic aid (EDTA) and incubated in 8.0 M urea for 4 h at 37°C. Urea was removed via dialysis against low ionic strength TBS (0.01 M Tris-HCl and 0.05 M NaCl, pH 7.3). Monomeric CRP concentration was determined by the BCA protein assay. The filtered solution was stored at 4 °C. pCRP was also dialyzed with TBS to remove sodium azide.

### Cell culture

ARPE-19, a spontaneously arising human retinal pigment epithelium cell line, was obtained from the American Type Culture Collection (ATCC®CRL-2302™). ARPE-19 (passages 15-20) were cultured in a 50:50 mixture of Dulbecco modified Eagle medium (DMEM) and Ham’s F12 (Biowest) supplemented with 10% fetal bovine serum (FBS, Biowest), 2 mM L-glutamine (Biowest), 100 U/mL penicillin (PAA), 0.1 mg/mL streptomycin (Biowest), and 1mM sodium pyruvate (Sigma) in a humidified incubator at 37°C in 5% CO_2_. Cells were passed every 4 to 6 days by trypsinization. ARPE-19 cells were plated at confluence onto semi-permeable polycarbonate Transwell® filters, 0.4 μm pore size. At day 3 FBS was reduced to 2 % and cells were maintained in a 37°C and 5% CO_2_ incubator for 2–4 weeks, changing media every 3–4 days.

Primary porcine RPE cells were isolated and cultured following the protocol described by [48] with some modifications. Eyes were trimmed of excess tissue and placed in 0.2% povidone iodine for 10 minutes on ice. Eyes were rinsed with sterile distilled water and placed in 1000 U/mL Penicillin-Streptomycin on ice for a minimum of 5 minutes. Anterior segments were removed with a scalpel at the ora serrata. Eyecups were filled with 1 mM EDTA and incubated at 37°C for 30 minutes to loosen the neural retina from the RPE sheet. The retina was gently pulled and detached from the RPE sheet. RPE cells were collected after incubation of the eyecups with 0.05% trypsin with 0.67 mM EDTA at 37°C. After trypsin inactivation with 10% FBS, RPE suspension was centrifuged and plated in DMEM High Glucose (Capricorn Scientific), with L-glutamine and sodium pyruvate, supplemented with 1% penicillin-streptomycin, 1% non-essential amino acids (Corning) and 10% FBS. At day 3 of culture, 5 μg/ml of ciprofloxacin (Sigma) was added to the medium and at day 7, serum was decreased to 1%. Cell monolayers were pigmented and showed the characteristic cobblestone morphology. At day 14, RPE cells were trypsinized and plated at confluence onto semi-permeable polycarbonate Transwell® filters, 0.4 μm pore size, previously coated with laminin. RPE cells were maintained in a 37°C and 5% CO_2_ incubator for 2–4 weeks and fed with 1% FBS growth medium every 3–4 days. These RPE cells expressed RPE-specific markers and showed high levels of TEER (see Supplementary Figure 1).

Choroidal endothelial cells from porcine eyes were isolated and cultured as described by Browning et al. [49] with few modifications. Briefly, after the retina and RPE cell layer were removed with a cell scraper, the complex choroid-Bruch’s membrane was peeled off from the sclera, cut into small pieces and washed three times with Minimum Essential Medium (MEM, ThermoFisher). The pieces were incubated with 0.1% collagenase (Sigma) for 2 hours at 37°C. The collagenase was neutralized with MEM containing 10% FBS and the mixture was passed through a 20G syringe. After centrifugation and washing with isolation medium, cells were resuspended in 0.1% BSA-PBS, adjusted to 1 x 10^7^ cells/ml, and incubated with rabbit anti-CD31 (Abcam) (20 μl per ml of cell suspension) for 1 hour at room temperature (RT) with agitation. Cells were centrifuged, washed with PBS and incubated with Dynabeads® for 45 minutes at 4°C. Endothelial cells positively selected were resuspended in EGM-MV2 (PromoCell) without hydrocortisone and seeded onto 0.5% gelatin coated wells. CECs expressed characteristic endothelial markers (CD31, and VWF) and showed the capacity of endothelial tube formation for up to passage 8 (see Supplementary Figure 2). CECs were plated at confluence onto semi-permeable polycarbonate Transwell® filters, 0.4 μm pore size, previously coated with 10 μg/mL fibronectin and maintained at 37°C and 5% CO_2_ incubator in EGM-MV2 media (Promocell).

### Measurement of transepithelial electrical resistance (TEER)

TEER was measured using a commercial electrical resistance system (Millicell; Millipore) in ARPE-19 and primary porcine RPE monolayers grown on Transwell filters as described above. TEER values were calculated by subtracting the value of a blank (transwell filter without cells). Measurements were repeated at least three times for each filter, and each experiment was repeated at least five times using 2 filters.

### Permeability assay

The paracellular permeability of ARPE-19 and primary porcine RPE monolayers was assessed by measuring the passive permeation of FITC-dextran (40 kDa, Sigma-Aldrich) across confluent cells grown on filters for a minimum of 3 weeks. Then, the RPE monolayers were treated with CRP isoforms (10 μg/mL) for 48h. After 48h treatment, 500 μg/ml FITC-dextran were added to the apical compartment of the chamber and samples (200 μl) from the basal medium (lower chamber) were collected 120 min after addition of FITC-dextran. The absorbance of basal and apical medium samples was measured at 485 nm of excitation and 528 nm of emission in a microplate reader (Infinite 200 PRO multimode, Tecan Group Ltd., Switzerland). Each condition was assayed in triplicate and repeated in at least five independent experiments. The diffusion rate was expressed as a percentage and calculated as follows: (amount of dextran lower chamber) x100 / (amount of dextran upper chamber).

### Immunofluorescence

The distribution of ZO-1 and RPE65 in RPE monolayers, CD31 and VWF expression in CECs, and CRP binding to RPE and CECs was examined by immunofluorescence. Filters were cut out, washed with PBS and fixed with 3.8% paraformaldehyde in PBS for 15 min at room temperature (RT). Cells were then washed with PBS, permeabilized with Triton X-100 (0.2% for ZO-1 and CD31, and 0.5 % for RPE65) for 15 minutes and blocked twice with filtered 1% BSA. Cells were then incubated with primary antibody anti-ZO-1 (clone 1A12, Thermo Scientific), anti-RPE65 (clone 401.8B11.3D9, Abcam), anti-CD31 (Abcam), anti-mCRP (3H12 gently provided by Dr LA Potempa) or anti-pCRP (1D6, gently provided by Dr LA Potempa) overnight. After washing three times with PBS, cells were incubated with secondary antibody Alexa Fluor anti-mouse 488 or 568 IgG or anti-rabbit 568 IgG for 1h at RT. Nuclei were counterstained with DAPI. Controls were stained with secondary antibodies only. Stained cells were washed and covered with Prolong Gold antifade reagent (Life Technologies). Images of immunostained cells were recorded on the high-speed spectral confocal microscope Leica TCS-SP5 and analyzed with ImageJ software. ZO-1 was intensity at the tight junctions (TJs) was measured as intensity at the intercellular junction divided by the intensity at the cytoplasm [50].

### SDS-PAGE and immunoblotting

The presence of CRP isoforms on apical and basolateral compartments was detected by SDS-PAGE and immunoblotting. Supernatants were centrifuged at 1,000 *g* for 10 minutes, loaded onto 12.5% polyacrylamide gels and run at 30 mA for 60 minutes. In order to avoid denaturalization of pCRP, samples were not heated and the amount of SDS in the acrylamide gels and the loading and electrophoresis buffers was reduced to 1/20 [51]. Proteins were transferred to a 0.22 μm nitrocellulose membrane performing a semi-dry transfer protocol. Non-specific binding sites were blocked with 5% non-fat dry milk in 0.1% PBS-Tween for 1 hour at RT, before incubation with anti-mCRP-specific monoclonal antibody 3H12 1:300 in blocking buffer ON at 4°C. Membranes were incubated with the secondary antibody linked to HRP (GAM-HRP, Bio-Rad) for 1 hour at RT. Chemiluminescent signal was detected with the Amersham ECL™ Prime Western blotting detection reagent (GE Healthcare) with ImageQuant LAS4000 (GE Healthcare) and bands were analyzed using ImageJ software.

### Determination of mCRP in cell supernatants

mCRP was detected in cellular supernatants (previously centrifuged at 1,000 g for 10 min) by an ELISA assay following the protocol recently described by Zhang et al. [52]. For this purpose, mouse anti-human CRP mAb CRP-8 (Sigma-Aldrich, C1688) was immobilized as capture antibody at 1:1,000 in coating buffer (10 mM sodium carbonate/bicarbonate, pH 9.6) overnight at 4°C. After washing three times for 2 minutes each with TBS, non-specific binding sites were blocked with filtered 1% BSA-TBS for 1 hour at RT. Samples diluted 1:100 in blocking buffer were added into wells for 1 hour at RT. Then, washing step was repeated and samples were incubated with sheep anti-human CRP polyclonal antibody (1:2,000 in blocking buffer) (BindingSite), prior incubation with a HRP-labeled donkey anti-sheep IgG (1:10,000 in blocking buffer) (Abcam). Signaling was detected with VersaMax Microplate Reader and The OD value of each sample was calculated as OD_450_–OD_570_ nm.

### Statistical analysis

Results were expressed as mean ± SD. Student’s *t* test or ANOVA followed by Dunnett’s posthoc analysis were used to determine statistical significance between treatments. A value of P<0.05 was considered significant. All calculations were performed using GraphPad Prism (GraphPad Software, San Diego, CA, USA).

## Supplementary Material

Supplementary Figures
