# Supplementary material for: Activation of C-reactive protein proinflammatory phenotype in the blood retinal barrier in vitro: implications for age-related macular degeneration
Source: Aging (Albany NY). 2020 Jul 16;12(14):13905–23. doi: 10.18632/aging.103655 (PMC7425453; doi:10.18632/aging.103655)
Supplement: Supplementary Figures [file aging-12-103655-s001..pdf]

SUPPLEMENTARY FIGURES

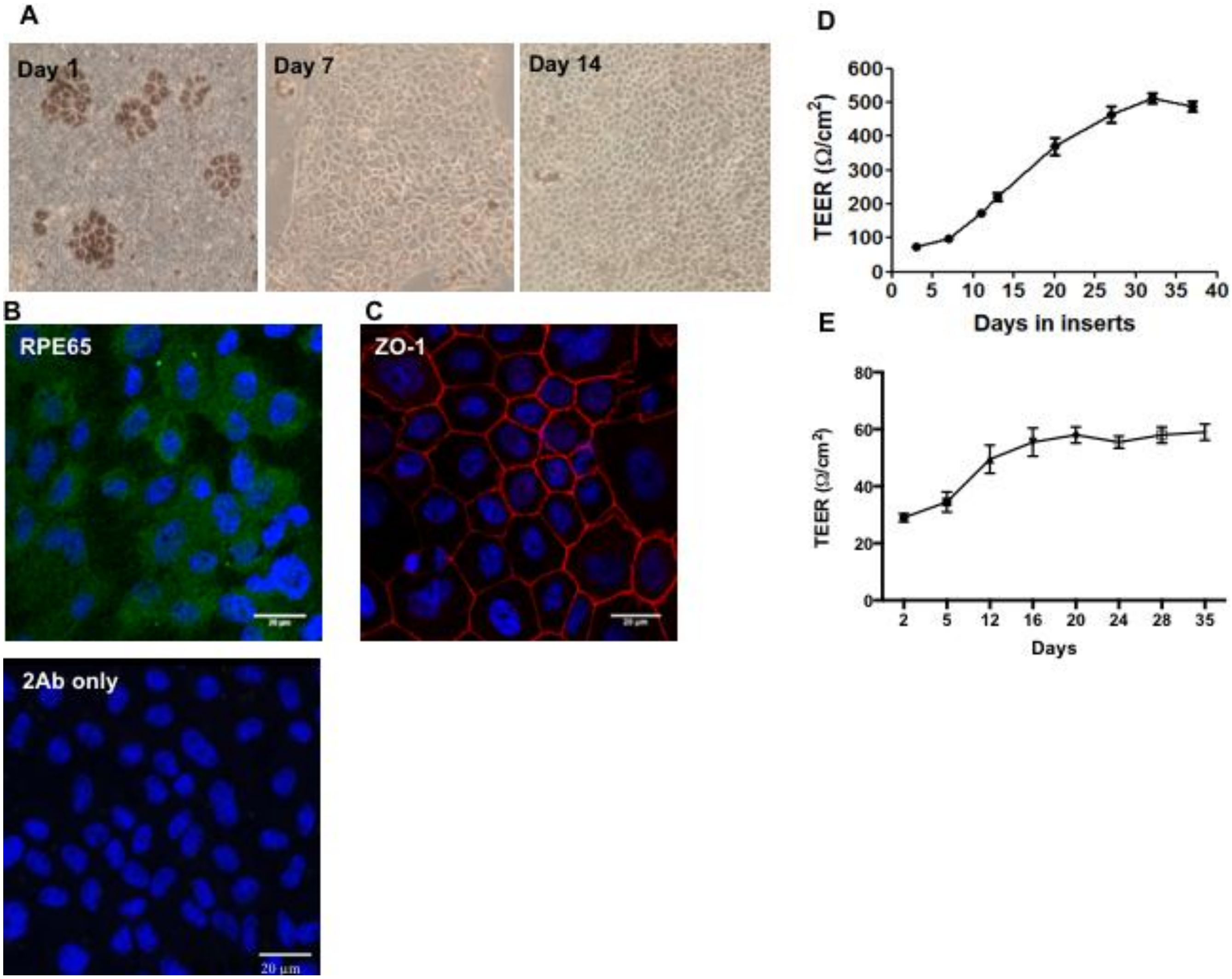

Supplementary Figure 1. Characterization of RPE cells. (**A**) Cells harvested for 1, 7 and 14 days after plating. Objective lens 10x. Primary porcine RPE cells cultured for 30 days were stained with antibodies to RPE65 (green) (**B**) and ZO-1 (red) (**C**). Scale bar = 20 µm. (**D**) TEER values of primary porcine RPE cells plated at 280,000 cells/cm^2 on laminin coated Transwell™ filters. (**E**) TEER values of ARPE-19 cells plated at 250,000 cells/cm^2 on Transwell™ filters for 35 days.

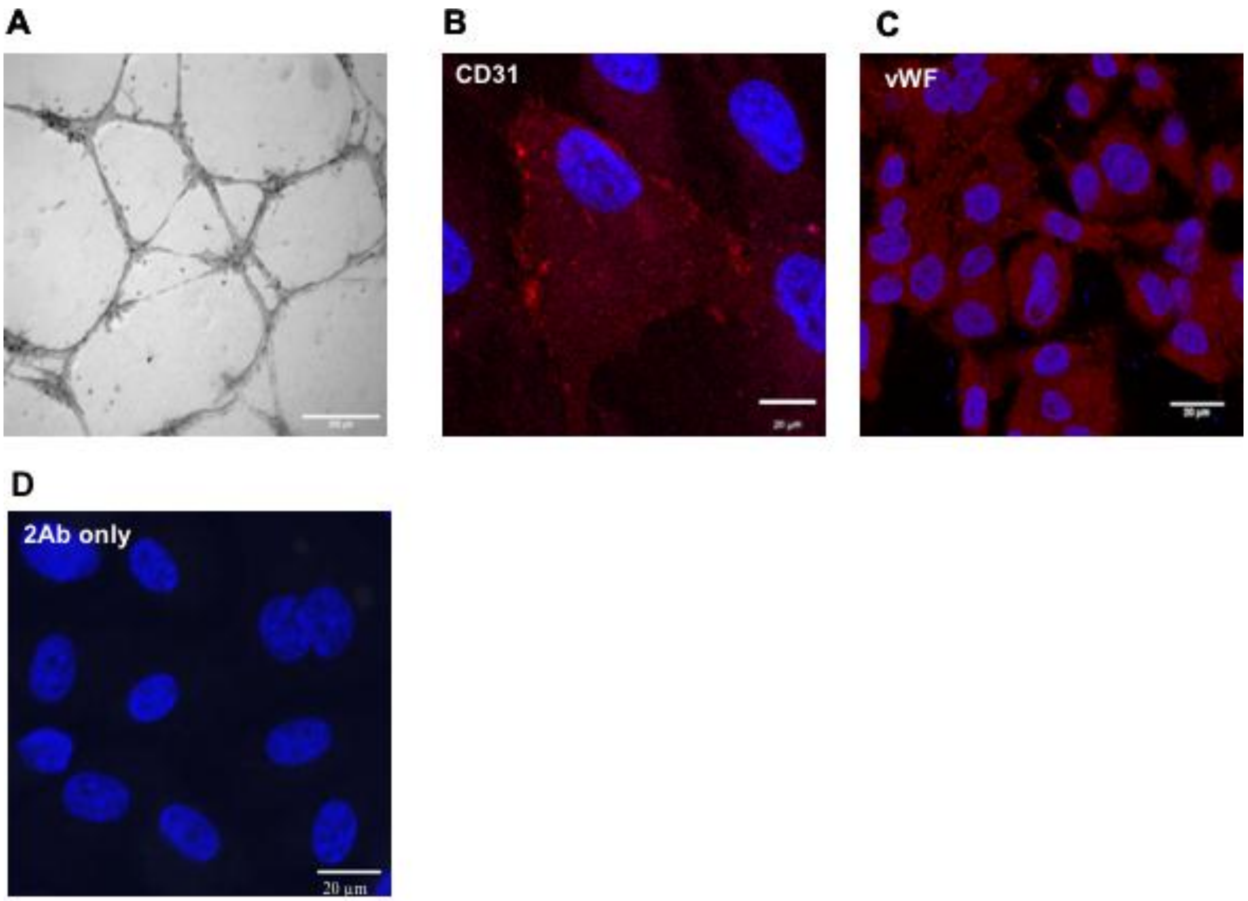

Supplementary Figure 2. Characterization of primary porcine CECs. (**A**) Primary porcine CECs were cultured into pure matrigel-coated wells and allowed to form capillary-like structures for 24 hours. Scale bar = 500 µm. Primary porcine CECs were stained with antibodies against CD31 (**B**) and VWF (**C**). Scale bar = 20 µm. (**D**) Negative control with cells stained without primary antibody.

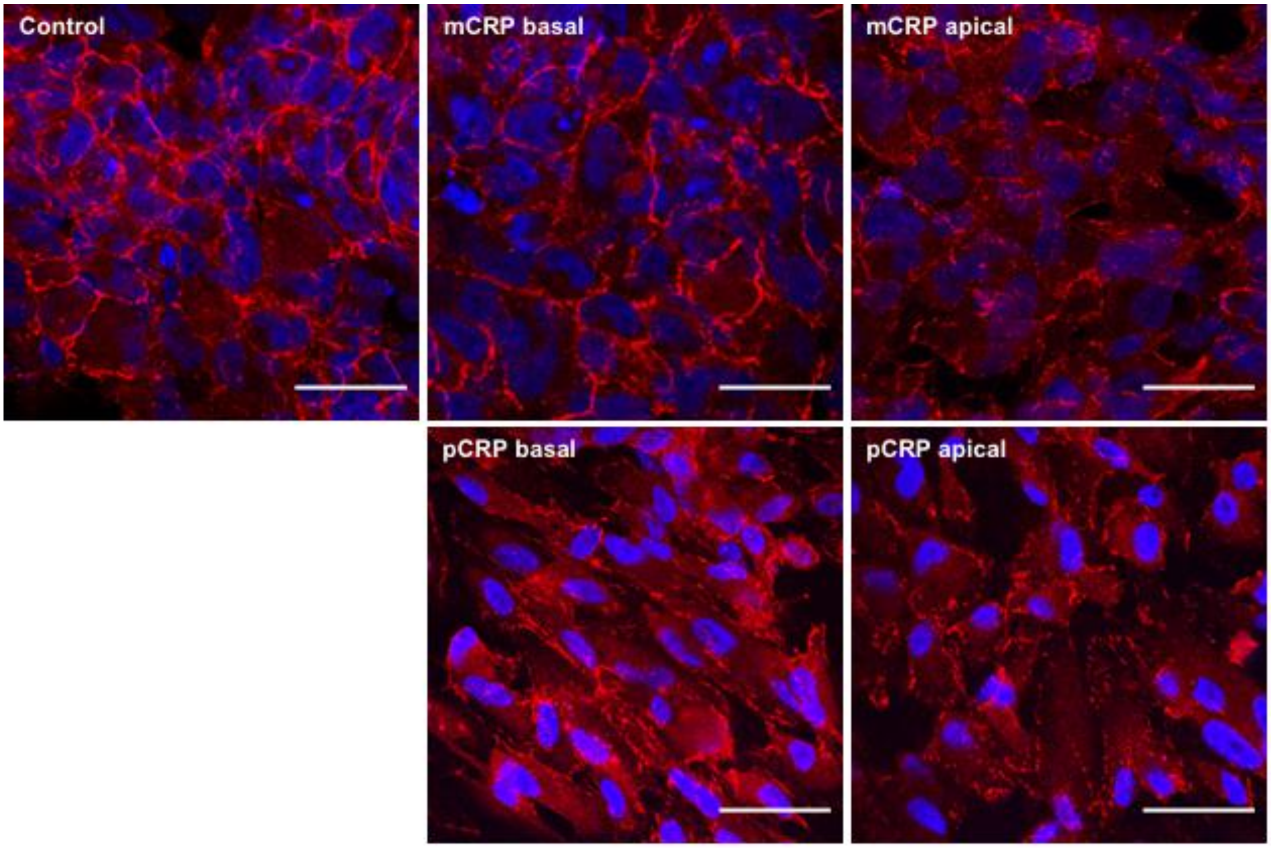

Supplementary Figure 3. Effect of CRP isoforms on ZO-1 expression in ARPE-19 cells. Cells were fixed and immunostained with anti ZO-1 (red) and DAPI (blue). Scale bar = 30 µm.

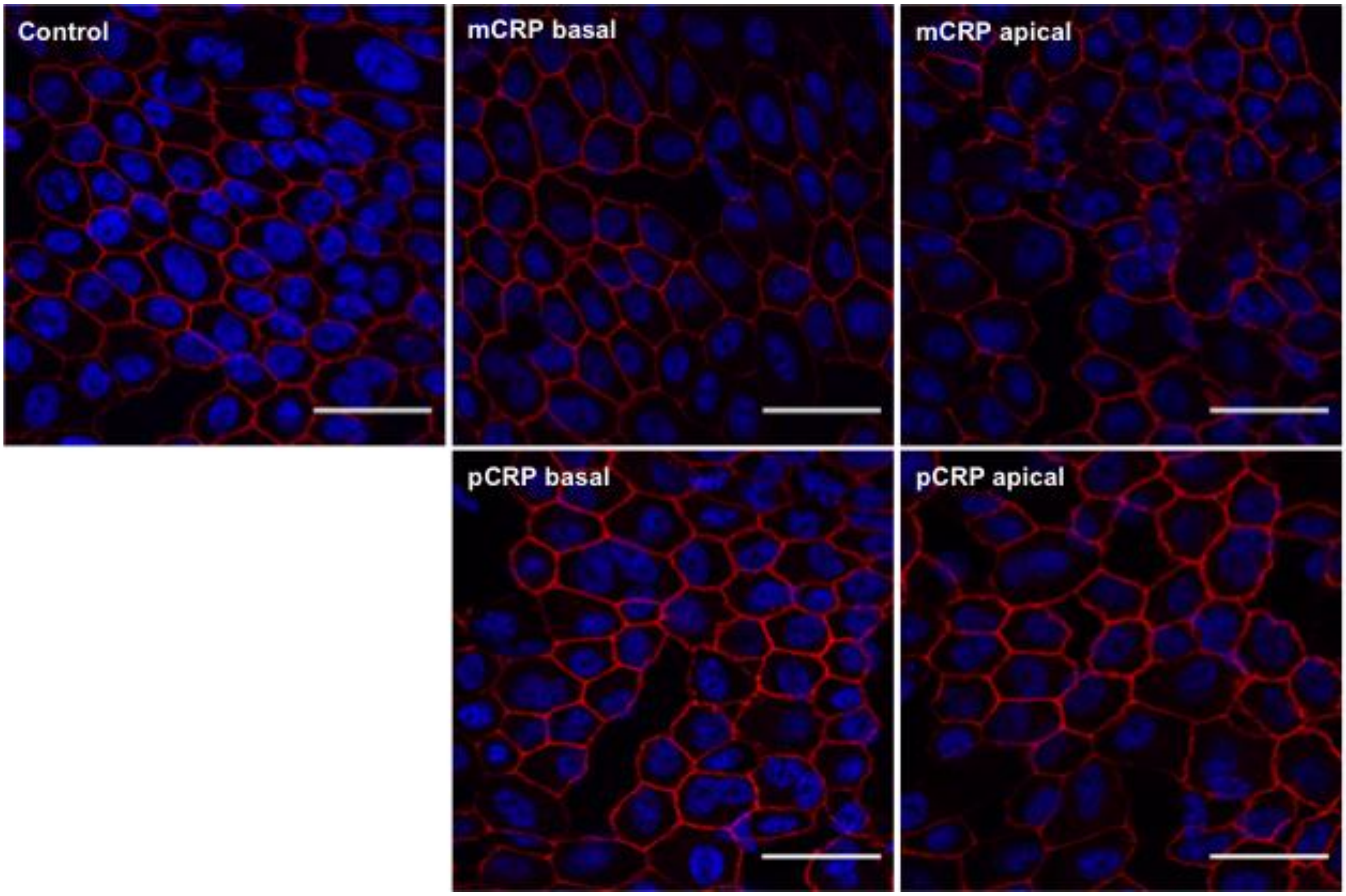

Supplementary Figure 4. Effect of CRP isoforms on ZO-1 expression in primary porcine RPE cells. Cells were fixed and immunostained with anti ZO-1 (red) and DAPI (blue). Scale bar = 30 µm.
